# Secondary Metabolites from the Marine Sponge Genus *Phyllospongia*

**DOI:** 10.3390/md15010012

**Published:** 2017-01-06

**Authors:** Huawei Zhang, Menglian Dong, Hong Wang, Phillip Crews

**Affiliations:** 1 School of Pharmaceutical Sciences, Zhejiang University of Technology, Hangzhou 310014, China; baixl2012@163.com (M.D.); yingzhoudengyuan@sina.com (H.W.); 2 Department of Chemistry & Biochemistry, University of California-Santa Cruz, Santa Cruz, CA 95064, USA; pcrews@ucsc.edu

**Keywords:** marine sponge, *Phyllospongia* sp., secondary metabolites, bioactivity

## Abstract

*Phyllospongia*, one of the most common marine sponges in tropical and subtropical oceans, has been shown to be a prolific producer of natural products with a broad spectrum of biological activities. This review for the first time provides a comprehensive overview of secondary metabolites produced by *Phyllospongia* spp. over the 37 years from 1980 to 2016.

## 1. Introduction

Marine sponges, as very primitive animals, are widely distributed in the oceans from tropic to polar regions. Growing evidence indicates that these animals are the most prolific source of natural products as pharmaceutical leads [1,2,3]. Marine sponges possess a large variety of secondary metabolites with diverse chemical structures, such as terpenoids [4], macrolides [5], and sterols [6]. Therefore, it has greatly attracted the attention of natural product chemists and pharmaceutical experts around the world to carry out chemical research for the new drug discovery.

*Phyllospongia* (Porifera, Demospongiae, Dictyoceratida, Thorectidae) is one of the most common marine sponges in tropical and subtropical areas, including the Indian Ocean, the Great Barrier Reef, Papua New Guinea, the South China Sea, the South Pacific, and the Red Sea. Chemical investigation of *Phyllospongia* spp. has been extensively carried out and has given rise to a great array of bioactive secondary metabolites. In order to better understand and rationally exploit the marine sponge genus *Phyllospongia*, relevant research references reported between 1980 and 2016 are summarized in this review for the first time.

## 2. Natural Products from *Phyllospongia* spp.

By 2016, a total of 132 various secondary metabolites (**1**–**132**) had been isolated and characterized from the marine sponge genus *Phyllospongia*, including *P. dendyi*, *P.* (syn. *Carteriospongia*) *foliascens*, *P. lamellosa*, *P. madagascarensis*, *P. papyracea*, *Carteriospongia* (syn. *Phyllospongia*) *flabellifera*, and other unidentified *Phyllospongia* spp. (Table 1). In terms of their chemical structures, most *Phyllospongia* sponge-derived natural products are sesterterpenoids, especially scalaranes [7], which are classified as C_25_ (scalarane), C_26_ (homoscalarane), and C_27_ (bishomoscalarane) [8]. Bioassay results indicated that some chemicals have pronounced biological activities and can be used as lead drugs. These secondary metabolites are summarized below according to biological origin.

### 2.1. Phyllospongia dendyi

**Chart 1:**
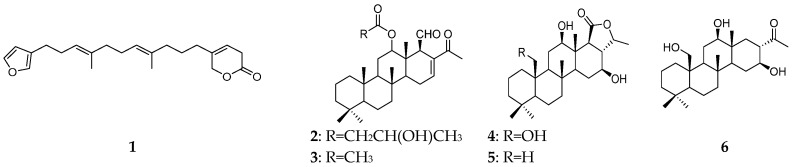

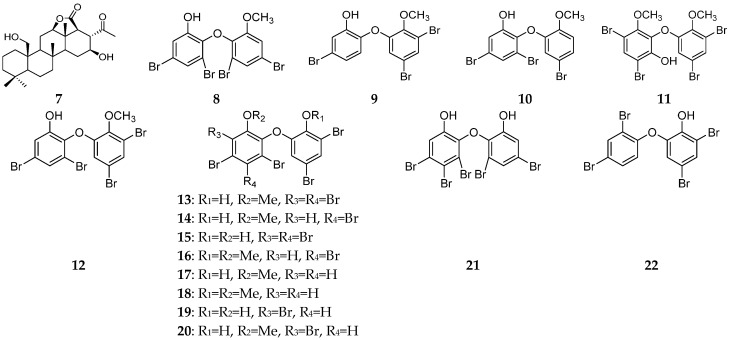
Chemical structures of secondary metabolites (**1**–**22**) from *P. dendyi*.

One new C_22_ furanoterpene, furodendin (**1**), was characterized from *P. dendyi* collected near Cairns on the Great Barrier Reef together with two known C_26_ tetracyclic sesterterpenes (**2**, **3**) in 1980 [9]. One specimen of *P. dendyi* collected from the coasts of Andaman and Nicobar Islands in the Indian Ocean was found to produce three novel scalaranes (**4**–**6**) and a known sesterterpene (**7**) [10]. Fifteen brominated compounds, bromophenols (**8**–**12**) [11] and diphenyl ethers (**13**–**22**) [12], were detected in *P. dendyi* grown at Palau Islands (Chart 1). To the best of our knowledge, *P. dendyi* is the only sponge that metabolizes brominated compounds among the *Phyllospongia* genus. Bioassay results suggested that these bromides have a broad spectrum of biological activities, including antimacroalgal activity [11], inhibitory effects on the assembly of microtubule proteins, and the meiotic maturation of starfish oocytes [12].

### 2.2. Phyllospongia (*syn.* Carteriospongia) foliascens

Marine sponge *P. foliascens* is the most productive maker of secondary metabolites among all known *Phyllospongia* spp. Chemical investigation indicates that most of these compounds belong to scalarane sesterterpenoid. *P. foliascens* grows in many marine areas, such as Okinawa, the South China Sea, Papua New Guinea, Indonesia, the South Pacific near Vanuatu, and the Great Barrier Reef. Interestingly, the same species of marine sponge collected from different areas possesses various scalarane sesterterpenoids.

**Chart 2:**
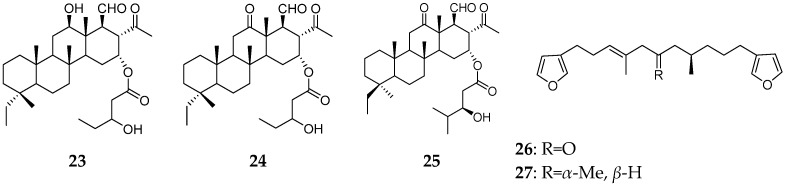
Chemical structures of secondary metabolites (**23**–**27**) from *P. foliascens*.

After Kikuchi et al. firstly reported the isolation of one novel anti-inflammatory scalarane bishomosesterterpene foliaspongin (**23**) from the Okinawan *P. foliascens* in 1981 [13], two new scalarane-type bishomosesterterpenes, dehydrofoliaspongin (**24**) and phyllofoliaspongin (**25**), and two new furanoterpenes, dihydrofurospongin-2 (**26**) and furospongin-1 (**27**), were also characterized from Okinawan *P. foliascens* (Chart 2). Compound **25** exhibited a broad spectrum of pharmacological effects, such as cytotoxic, anti-thrombocyte, and vasodilative activities [14].

**Chart 3:**
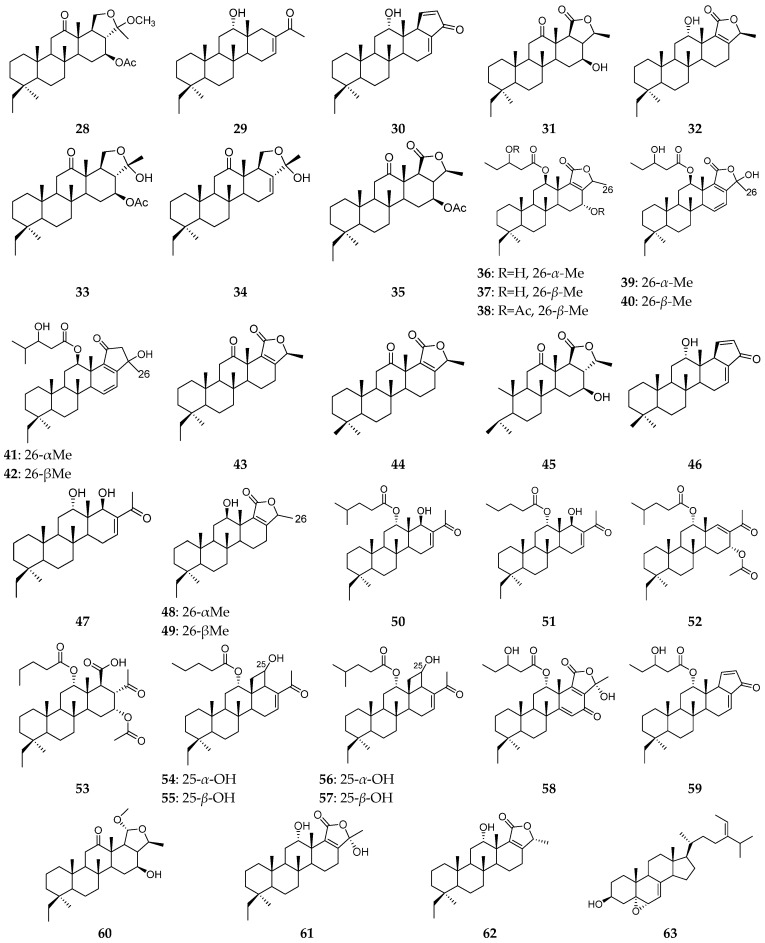
Chemical structures of secondary metabolites (**28**–**63**) from *P. foliascens*.

Natural products from the marine sponge *P. foliascens* collected from the South China Sea are abundant. Since 1989, up to 36 compounds have been reported. In 1991, Fu and his coworkers isolated and identified 7 compounds: phylloketal (**28**) [15], phyllofenone A (**29**) [16], phyllofenone B (**30**), phyllofolactones A–B (**31**–**32**) [17], and phyllohemiketal A-B (**33**–**34**) [18]. Among these secondary metabolites, compound **29** showed weak antifungal activity against *Candida pseudotropicalis*, while compound **30** displayed cytotoxic activity against the P388 murine leukemia cell line with an IC_50_ value of 5 μg/mL. Acetoxy phyllofolactone A (**35**) [19] and phyllactones A–E (**36**–**40**) [20] were found and characterized in 1992. Compounds **36** and **37** had moderate in vitro cytotoxicity against KB cells with the same IC_50_ value of 20 μg/mL. Interestingly, phyllactones F–G (**41**–**42**) [21] and phyllofolactones C–D (**43**–**44**) [22] were also detected in the same specimen. Phyllofolactone L (**45**) and phyllofenone D–E (**46**–**47**) belong to ascalarane sesterterpenoids [23]. Their chemical structures were elucidated on the basis of spectroscopic analysis. A biological assay showed that only compound **47** had moderate cytotoxic activity against the leukemia cell line P388, with an IC_50_ value of 6.5 μg/mL. Two new sesquiterpenes, phyllofolactone F (**48**) and phyllofolactone G (**49**), together with phyllofenone D (**46**) and phyllofenone E (**47**), were isolated and identified by chromatographic methods and modern analytical methods [7]. Carteriofenones A–C (**50**–**52**) were 20,24-bishomo-25-norscalaranes and carteriofenones D–K (**53**–**60**) and one analogue (**61**) belong to 20,24-bishomoscalaranes [24]. Moreover, phyllofolactone M (**62**) and a new sterol, (24*E*)-5*α*,6*α*-epoxystigmasta-7,24(28)-dien-3*β*-ol (**63**), together with a known sesterterpene, phyllofolactone B (**32**), were detected in the same sample collected from the South China Sea [25] (Chart 3).

**Chart 4:**
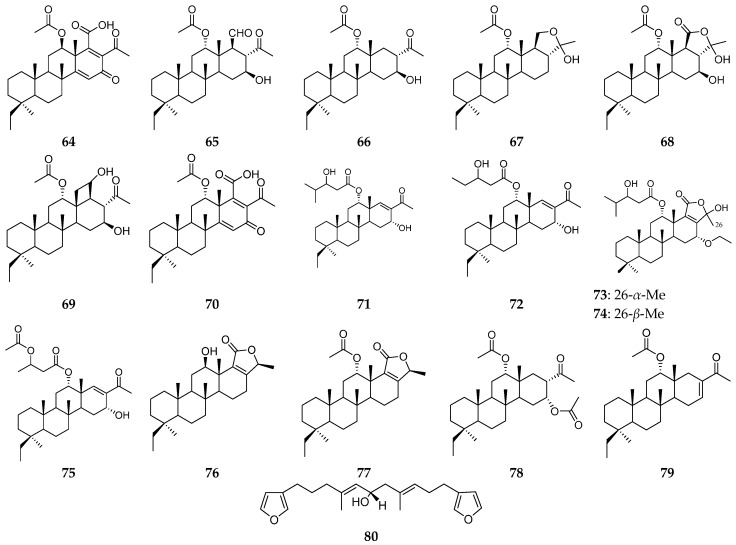
Chemical structures of secondary metabolites (**64**–**80**) from *P.* (syn. *Carteriospongia*) *foliascens*.

Seven 20,24-dimethylscalarane derivatives (**64**–**70**) were characterized from *P.* (syn. *Carteriospongia*) *foliascens* gathered from Papua New Guinea. Compounds **64** and **70** were C-14 anomers [26,27]. Chemical investigation of *P.* (syn. *C.*) *foliascens* collected from the Indonesian sea afforded five scalarane sesterterpenoids (**71**–**75**), which possessed Ras Converting Endoprotease (RCE) inhibitory effect except **72** [28]. Specimens of *P.* (syn. *C.*) *foliascens* from the South Pacific could metabolite 12-*epi*-phyllofolactone B (**76**) [29], while the sample collected from the Great Barrier Reef produced scalarane sesterterpenoids (**77**–**79**) [30,31]. Additionally, one furanoterpene (**80**) was also isolated from the marine sponge *P.* (syn. *C.*) *foliascens* [32] (Chart 4).

### 2.3. Phyllospongia lamellosa

**Chart 5:**
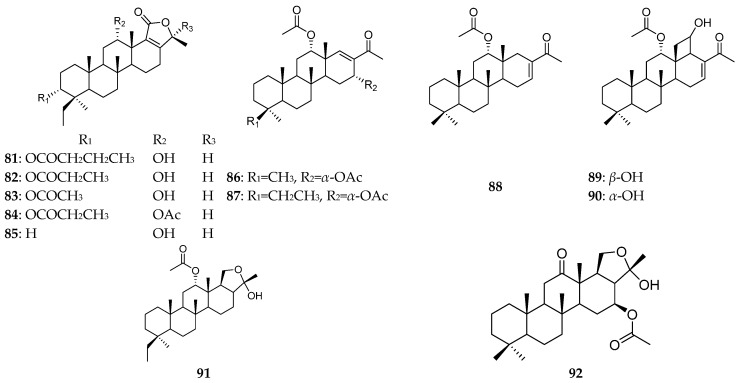
Chemical structures of secondary metabolites (**81**–**92**) from *P. lamellosa*.

Until now, there have only been two reports on the chemical study of the marine sponge *P. lamellosa* in the database Web of Sciences [33]. Five 20,24-bishomoscalane sesterterpenes, phyllolactones A–E (**81**–**85**), were characterized from *P. lamellosa* collected in the Indo-West Pacific Ocean. Bioassay results indicated that these secondary metabolites possessed an inhibitory effect on human immunodeficiency virus type 1 (HIV-1) envelope-mediated fusion in vitro with IC_50_ values of about 2 μM [34]. From the same marine sponge derived from the Egyptian Red Sea, five new scalarane sesterterpenes, phyllospongins A–E (**86**–**90**), were detected together with four known derivatives (**76**, **91**, **92** and **99**) [8] (Chart 5). Compounds **87**–**91** had potent cytotoxic activity against HCT-116 as the positive control doxorubicin, while **90** showed cytotoxic activity against MCF-7.

### 2.4. Phyllospongia madagascarensis

**Chart 6:**
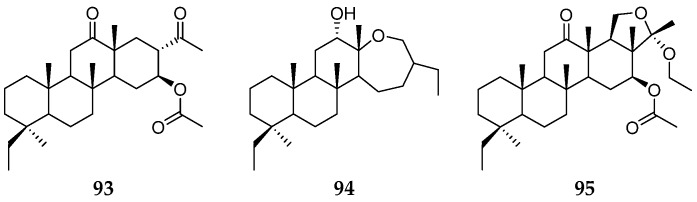
Chemical structures of secondary metabolites (**93**–**95**) from *P. madagascarensis*.

To the best of our knowledge, only three natural products (**93**–**95**) (Chart 6) have been found in the marine sponge *P. madagascarensis*, which was grown near the northwest coast of Madagascar [35]. Interestingly, the chemical structure of **94** possesses seven-membered oxacycle.

### 2.5. Phyllospongia papyracea

**Chart 7:**
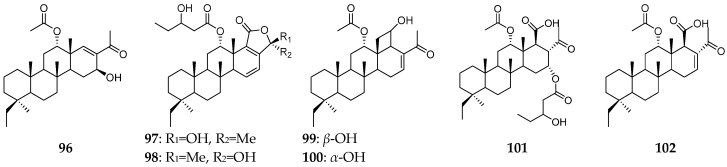

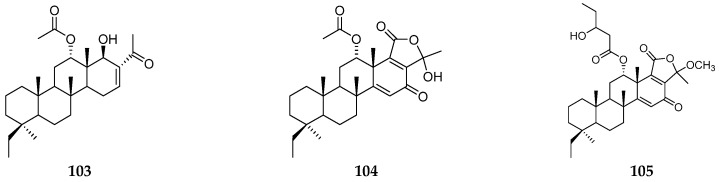
Chemical structures of secondary metabolites (**96**–**105**) from *P. papyracea*.

Ten small molecules (**96**–**105**) (Chart 7) were isolated from *P. papyracea* collected on Hainan Island in the South China Sea, Papua New Guinea, and Sangihe Island in the Indonesian Sea [36,37,38]. Cytotoxic tests suggested that compound **96** had an in vitro cytotoxic effect on the leukemia cancer cell line P388 with an IC_50_ value of 5 μg/mL, and **99**–**104** were inactive against the *β*-catenin and transcription factor 4 (Tcf4) complex. Phyllactone H (**105**) was found in the marine sponge derived from Sangihe Island and possessed in vitro moderate cytotoxicities against cell lines A549, MCF-7, and HeLa with IC_50_ values of no more than 25 μM.

### 2.6. Carteriospongia (*syn.* Phyllospongia) flabellifera

**Chart 8:**
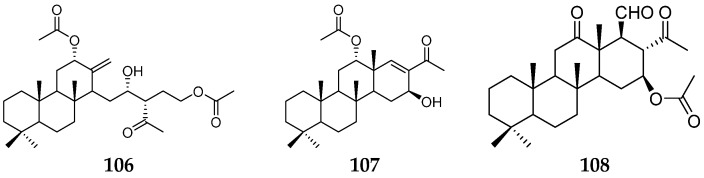
Chemical structures of secondary metabolites (**106**–**108**) from *C.* (syn. *Phyllospongia*) *flabellifera*.

The marine sponge *C.* (syn. *P.*) *flabellifera* is usually distributed in a wide corridor of the Indo-Pacific Ocean. A chemical study of the marine sponge collected in the South Pacific Ocean led to the isolation of two new small molecules, flabelliferins A (**106**) and B (**107**) [39]. Compound **106** had a rare 25-homocheilanthane carbon skeleton, and **107** exhibited inhibitory effect on the human colon tumor cell lines KM12 and COLO205. A new sesterterpenoid derivative (**108**) was also characterized from *C. flabellifera* collected around the Great Barrier Reef [40] (Chart 8).

### 2.7. Other Phyllospongia *spp.*

**Chart 9:**
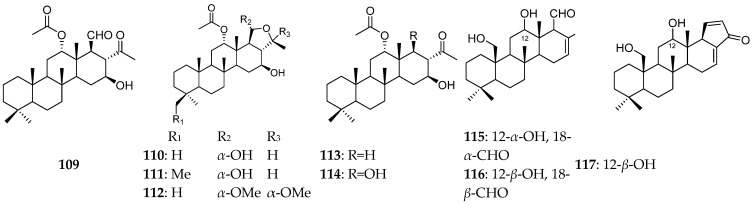

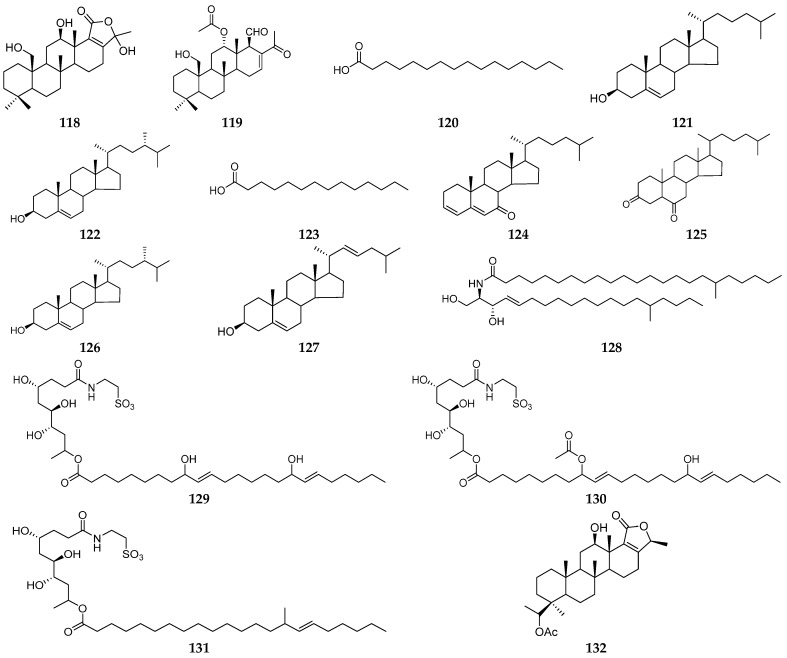
Chemical structures of secondary metabolites (**109**–**132**) from other *Phyllospongia* spp.

Up to 24 secondary metabolites (**109**–**132**) (Chart 9) were isolated and identified from other unclassified *Phyllospongia* spp. Compounds **109**–**114** from an Indonesian marine sponge displayed 30%–95% inhibition of the growth of KB cells at 10 μg/mL [41]. Compounds **115**–**119** were produced by the *Phyllospongia* sp. collected from Northern Madagascar. Compounds **115**, **116**, and **119** possessed strong in vitro cytotoxic activities against human ovarian cancer cell line A2780 with IC_50_ values of 0.26, 0.28, and 0.65 μM, respectively, while **117** and **118** had moderate activities with IC_50_ values of 4.5 and 8.7 μM, respectively. Compound **116** exhibited a strong inhibitory effect on the human lung non-small cell line H522-T1 with an IC_50_ value of 0.61 μM [42]. Compounds **120**–**127** [43] and phylloamide A (**128**) [44] were also isolated from the South China Sea sponge. Carteriosulfonic acids A–C (**129**–**131**) [45] and **132** [46] were respectively isolated from two specimens collected at Philippines and Fiji. Bioassay results suggested that **128**–**130** had an inhibitory effect on the growth of glycogen synthase kinase-3β (GSK-3β), with IC_50_ values of 12.5, 6.8, and 6.8 μM, respectively.

## 3. Conclusions

Natural products from the marine sponge genus *Phyllospongia* have been well studied over the last 37 years. Their frameworks are diverse, including terpenoid, macrolide, sterol, and ceramide. Moreover, the most common structure is sesterterpenoid, which has diverse biological activities. With the increasing development of oceanographic technology leading to the isolation of new *Phyllospongia* species from marine environments, more secondary metabolites with novel chemical structure(s) and/or potent bioactivities will be found in the near future.

## Figures and Tables

**Table 1:** *Phyllospongia* sponge-derived natural products.

Organism	Locality	Compound	References
*Phyllospongia dendyi*	the Great Barrier Reef	furodendin (**1**), sesterterpenes (**2**, **3**)	[9]
	Andaman and Nicobar Islands	scalaranes (**4**–**6**), sesterterpene (**7**)	[10]
	Palau Islands	bromophenols (**8**–**12**) and diphenyl ethers (**13**–**22**)	[11,12]
*P.* (syn. *Carteriospongia*) *foliascens*	the Okinawa	foliaspongin (**23**), dehydrofoliaspongin (**24**), phyllofoliaspongin (**25**), dihydrofurospongin-2 (**26**), furospongin-1 (**27**)	[13,14]
	the South China Sea	phylloketal (**28**), phyllofenone A (**29**), phyllofenone B (**30**), phyllofolactones A–B (**31**–**32**), phyllohemiketals A (**33**) and B (**34**), acetoxy phyllofolactone A (**35**), phyllactones A–G (**36**–**42**), phyllofolactones C–D (**43**–**44**), phyllofolactone L (**45**), phyllofenone D–E (**46**–**47**), phyllofolactone F–G (**48**–**49**), carteriofenones A-K (**50**–**60**), analogue (**61**), phyllofolactone M (**62**), sterol (**63**)	[7,15,16,17,18,19,20,21,22,23,24,25]
	Papua New Guinea	20,24-dimethylscalaranes (**64**–**70**)	[26,27]
	the Indonesia Sea	scalaranes (**71**–**75**)	[28]
	the South Pacific	12-*epi*-phyllofolactone B (**76**)	[29]
	the Great Barrier Reef	scalaranes (**77**–**79**)	[30,31]
	unknown	furanoterpene (**80**)	[32]
*P. lamellosa*	the Indo-West Pacific Ocean	phyllolactones A–E (**81**–**85**)	[33]
	the Egyptian Red Sea	phyllospongins A–E (**86**–**90**), sesterterpenes (**76**, **91**, **92**)	[8]
*P. madagascarensis*	Northern Madagascar	sesterterpenes (**93**–**95**)	[34]
*P. papyracea*	Hainan Island	scalaranes (**96**–**98**)	[35]
	Papua New Guinea	bishomoscalarane sesterterpenes (**99**–**104**)	[36]
	Sangihe Island	phyllactone H (**105**)	[37]
*C.* (syn. *P.*) *flabellifera*	the South Pacific Ocean	flabelliferins A (**106**) and B (**107**)	[38]
	the Great Barrier Reef	sesterterpenoid (**108**)	[39]
Unclassified *P.* spp.	the Indonesia Sea	scalaranes (**109**–**114**)	[40]
	Northern Madagascar	homoscalarane sesterterpenes (**115**–**119**)	[41]
	the South China Sea	fatty acids (**120**, **123**), steroids (**121**, **122**, **124**–**127**), phylloamide A (**128**)	[42,43]
	Philippines	carteriosulfonic acids A–C (**129**–**131**)	[44]
	Fiji	sesterterpene (**132**)	[45]
